# Tumor markers level profile in dermatomyositis, systemic sclerosis, systemic lupus erythematosus, rheumatoid arthritis and ovarian cancer

**DOI:** 10.1186/s40001-025-02892-x

**Published:** 2025-07-14

**Authors:** Jin-chi Cai, Lan Yang, Ying-sen Xue, Song-bo Wang, Li Zhang, Ke-nan Duan, Zhao-wei Gao, Jun Li

**Affiliations:** 1 Department of Orthopedics, Xi’an People’s Hospital (Xi’an Fourth Hospital), Xi’an, 710004 Shaanxi China; 2 Department of Clinical Laboratory, Tangdu Hospital, Air Force Medical University, Xi’an, 710038 Shaanxi China

**Keywords:** Dermatomyositis, Systemic sclerosis, Systemic lupus erythematosus, Rheumatoid arthritis, Ovarian cancer, Tumor markers

## Abstract

**Background:**

Autoimmune diseases (AID) have been showed to be susceptibility to malignancy. This study aimed to analyzed the profile of serum tumor markers in four common autoimmune disease.

**Methods:**

Patients with dermatomyositis (DM, *n* = 132), Systemic sclerosis (SSc, *n* = 77), Systemic lupus erythematosus (SLE, *n* = 191), Rheumatoid arthritis (RA, *n* = 160) and ovarian cancer (*n* = 250) were included in this study. Twelve tumor markers (CA724, AFP, FRT, NSE, CA19-9, CA125, CYFRA21-1, CA153, *β*-HCG and HE4) levels and abnormal rate in these patients were retrospective statistics. The tumor markers profiles were compared among the different AID.

**Results:**

Compared with ovarian cancer (OV) patients, there were no significant differences for the levels and abnormal rates of CYFRA21-1/HE4/CA50/FRT in AID patients. The levels and abnormal rates of CA724/FRT/CA125/NSE were higher in OV patients than that in AID patients. 75% AID patients have at least one elevated tumor marker. 69.46% AID patients have 2–5 elevated tumor markers. All the 12 tumor markers were negative in 16.67, 19.74, 27.23 and 32.70% of DM, SSc, SLE and RA patients. Except CA50, the levels of the other eleven tumor markers were significantly different between DM/SSc/SLE/RA. Except AFP/β-HCG/SCC, the abnormal rate of the other tumor markers were significantly different between these AID.

**Conclusions:**

The increased levels of tumor makers were common in four major AID, and the profile of tumor makers were significantly different among these AID.

## Introduction

Detection of serum tumor markers play an important role in screening, early diagnosis and prognosis monitoring of the cancer patients. Therefore, the accuracy of tumor markers determination is very important for clinicians’ judgment. In clinical examination, in addition to the intrinsic influencing factors of the tumor (i.e., tumor size, tumor site, tumor grade and stage, etc.), the level of tumor markers are also affected by many other factors, including sample hemolysis, drug use, detection methods, and other diseases, including autoimmune diseases [1].

Autoimmune diseases (AID) are a group of diseases with unknown etiology, characterized by autoimmune inflammation and autoantibodies production. Immune system disorder plays important roles in AID progression. Due to the immune tolerance deficiency, multiple tissues and organs are attacked by immune system and may cause other secondary diseases [2]. Recent studies have found that there is a correlation between autoimmune diseases and cancers [3–5]. First, patients with various AID are susceptibility to specific cancers [6]. On the other hand, cancer patients also produce a lot of autoantibodies, which were known as tumor autoantibodies [7, 8]. Recently, cancer susceptibility and incidence in AID have attracted much attention. Several theories have been proposed to explain the occurrence of cancers together with AIDs. First, Ami’s research found there is a close temporal relationship between the onset of cancer and scleroderma in patients with antibodies to RNA polymerase I/III [9]. Second, immunosuppressive therapy for autoimmune disease could account for the increase in malignancy risk in a subset of patients [10]. Chronic inflammation and repair process may predispose cells to malignant transformation [9]. Third, Cancers arise as a consequence of target tissue damage from the autoimmune disease, or from the cytotoxic therapies used to treat aggressive disease, or as a result of a defective immune system that predisposes an individual to developing both autoimmunity and cancer [11, 12]. To further explore the relationship between AID and cancers, in this study, we analyzed the profile of gynecological tumor markers in AID patients, including dermatomyositis (DM), systemic sclerosis (SSc), systemic lupus erythematosus (SLE) and rheumatoid arthritis (RA), and compare the differences between AID and ovarian cancer.

## Methods

### Subjects

We retrospectively analyzed tumor markers data of female patients with ovarian cancer [*n* = 250; age: 56.5 (IQR: 49–63)] and different autoimmune diseases (AID), including DM [*n* = 132; age: 54 (IQR: 45.75–61.25)], SSc [*n* = 77; age: 54 (IQR: 47–62)], SLE [*n* = 191; age: 37 (IQR: 31–49)] and RA [*n* = 160; age: 58 (IQR: 49.75–65)]. Patients were initially admission at the Tangdu Hospital, Air force Medical University from January 2021 to July 2023. The patients included in the study were recently diagnosed and had no prior medical history of cancer, smoking habits, family history of cancer, or any other known risk factors associated with cancer. The patients of DM fulfilled the diagnostic criteria outlined in the 2017 European League against Rheumatism (EULAR)/American College of rheumatology (ACR) classification criteria for adult and juvenile idiopathic inflammatory myopathies and their major subgroups [13]. The patients of SSc fulfilled the diagnostic criteria outlined in the 2013 classification criteria for systemic sclerosis: an EULAR/ACR collaborative initiative [14]. The patients of SLE fulfilled the diagnostic criteria outlined in the 2019 EULAR/ACR classification criteria for Systemic Lupus Erythematosus [15]. The patients of RA fulfilled the diagnostic criteria outlined in the 2010 rheumatoid arthritis classification criteria: an EULAR/ACR collaborative initiative [16]. Furthermore, all OV patients were confirmed by pathological examination. This study was approved by the Ethics Committee of Tangdu Hospital, Fourth Military Medical University, and the informed consent was waived for this study due to its observational nature and minimal risk to participants.

### Tumor marker detection assay

In total, 12 tumor markers were analyzed in this study, including cancer antigen 724 (CA724), alpha fetoprotein (AFP), ferritin (FRT), Neuron specific enolase (NSE), cancer antigen 19–9 (CA19-9), cancer antigen (CA125), cytokeratin 19 fragment antigen21-1 (CYFRA21-1), cancer antigen 153 (CA153), human chorionic gonadotropin (*β*-HCG), human epididymis (HE4), cancer antigen (CA50) and squamous cell carcinoma antigen (SCC). CA724, AFP, FRT, NSE, CA19-9, CA125, CYFRA21-1, CA153, *β*-HCG and HE4 were measured based on electrochemical luminescence assay adapted to an automated chemiluminescent analyzer (cobas8000, Roche). CA50 and SCC were detected by diagnostic kit (Savant Biotechnology Co., Ltd, Beijing) adapted to smart-3000 analyzer (Keysmile Biological Technology Co., Ltd, Chongqing).

### Statistical analysis

Shapiro–Wilk normality test was used to analyze the normality of the data. The tumor markers levels do not conform to a normal distribution, and were expressed as median (interquartile range, IQR). Wilcoxon’s test was used to evaluate the differences between different groups. Kruskal–Wallis test was used to analyze the differences among multiple groups. Spearman correlation analysis was used to analyze the correlation among tumor markers. The chi-square test and Fisher-exact test were used to analyze the differences in the abnormal rates of tumor markers between groups. All statistical analysis were performed by R (version 4.1.3) software. *P* value less than 0.05 was considered to indicate statistical significance.

## Results

### The differences of tumor makers between autoimmune diseases and OV

We analyzed the tumor marker levels in autoimmune disease and OV. As shown in Table 1. There were no significant differences of five tumor markers (i.e., CYFRA21-1, HE4, CA50, SCC and AFP) levels between autoimmune disease and OV patients. The other seven markers (i.e., CA72-4, FRT, NSE, CA19-9, CA125, CA153 and *β*-HCG) were significant higher in OV patients than that in AID patients. Furthermore, we analyzed the abnormal rates of these markers in AID and OV patients. The results showed that the positive rates of serum CA724, FRT, NSE and CA125 were higher in OV patients than in AID patients, while the positive rate of serum SCC were lower in OV patients than in AID patients. Notably, there were no statistically significant differences in the abnormal rates of AFP, CA19-9, CYFRA21-1, *β*-HCG, CA153, HE4 and CA50 between AID and OV patients.

**Table 1:** Difference of tumor markers between AID and OV patients

Tumor marker (reference interval)	Tumor marker levels [Median (IQR)]			Abnormal rate of tumor marker (%)			
	AID	OV	P	AID	OV	χ^2^	P
CA72-4 (0–6.9 U/mL)	1.795 (1.50–5.253)	3.460 (1.643–9.155)	< 0.001	19.11	33.20	44.012	< 0.001
AFP (0–7 ng/mL)	2.285 (1.60–3.170)	2.485 (1.685–3.493)	0.05	2.86	4.40	0.843	0.357
FRT (13–150 μg/L)	121.00 (50.00–258.50)	202.0 (102.2–408.8)	< 0.001	41.79	64.80	35.721	< 0.001
NSE (0–16.3 ng/mL)	11.80 (9.71–15.04)	14.10 (11.50–18.55)	< 0.001	17.86	34.40	25.811	< 0.001
CA19-9 (0–27 U/mL)	9.055 (5.138–15.100)	10.50 (6.567–18.900)	0.006	10.71	13.60	1.136	0.287
CA125 (0–35 U/mL)	14.025 (9.543–25.900)	30.80 (12.12–232.25)	< 0.001	18.75	46.40	65.217	< 0.001
CYFRA21-1 (0–3.3 ng/mL)	2.250 (1.650–3.280)	2.305 (1.640–3.72)	0.17	24.46	30.80	3.251	0.071
CA153 (0–26.4 U/mL)	11.600 (8.107–16.948)	18.25 (10.70–32.08)	< 0.001	7.50	7.60	< 0.001	1
*β*-HCG (0–7 U/mL)	0.645 (0.200–1.900)	1.085 (0.473–2.060)	< 0.001	1.96	0.40	1.925	0.118
HE4 (0–105 pmol/L)	83.25 (59.97–134.25)	74.15 (53.40–162.25)	0.33	37.68	36.80	0.026	0.873
CA50 (< 20 U/mL)	6.945 (4.558–10.457)	6.665 (3.993–9.953)	0.17	6.25	7.20	0.123	0.725
SCC (< 1.2 ng/mL)	0.5400 (0.410–0.730)	0.535 (0.42–0.70)	0.46	6.96	2.40	6.020	0.014

### Tumor marker levels pattern in patients with different AID

**Fig. 1:**
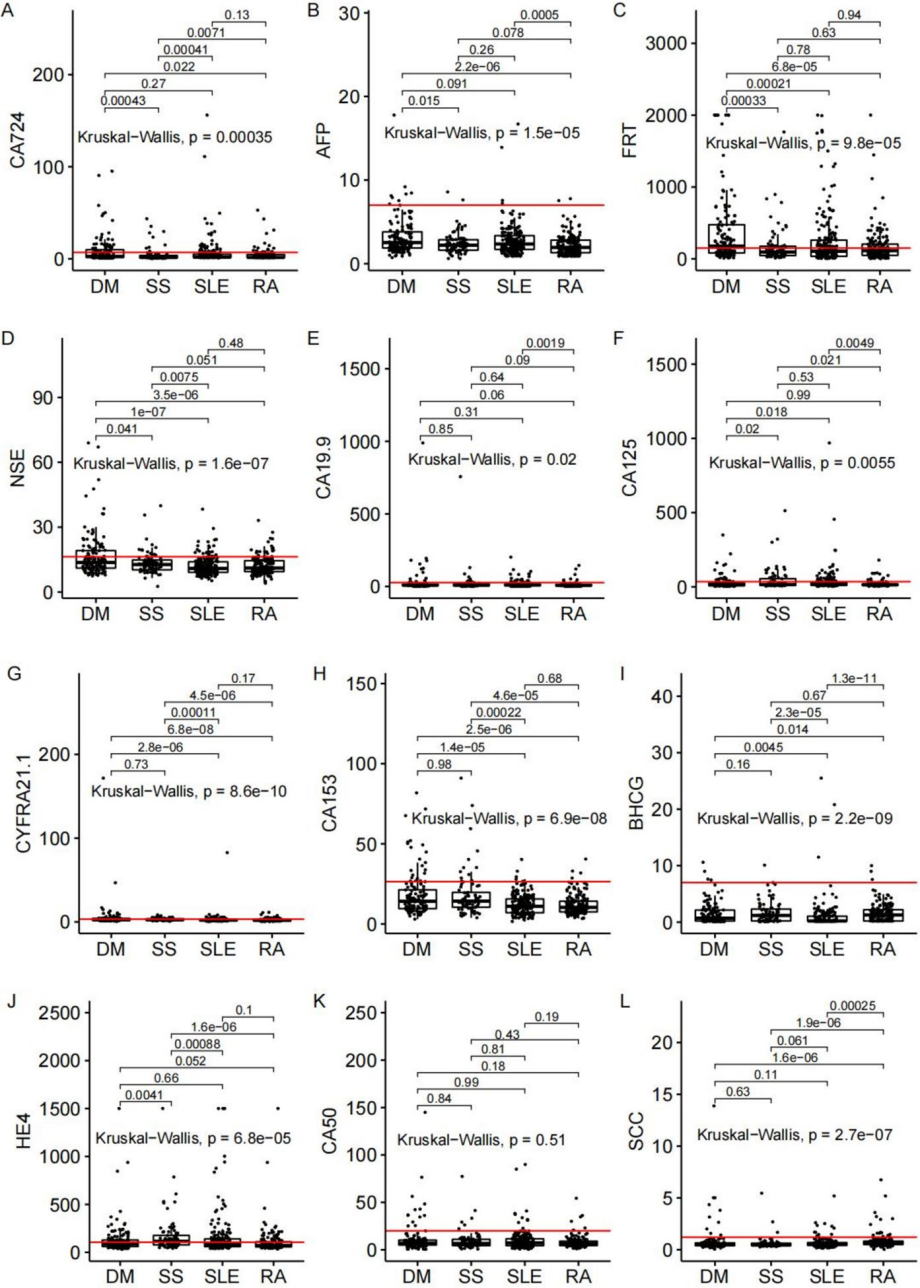
The levels of serum tumor markers in patients with DM, SSc, SLE and RA. (A-L) Differential analysis of CA724, AFP, FRT, NSE, CA19-9, CA125, CYFRA21-1, CA153, β-HCG, HE4, CA50 and SCC. Read line: the normal reference value of tumor markers

We analyzed the tumor marker levels and the differences in DM, SSc, SLE and RA patients. As shown in Fig. 1, first, except CA50, there were significant differences of the other tumor markers among the different AID patients. Second, in four types of AID patients, a certain proportion of patients had serum tumor marker levels higher than the reference interval (Table 2). Overall, the abnormal rates of CA724, FRT, NSE, CA125, CYFRA21-1, CA153 and HE4 were significant differences among four groups of AID patients (Table 2). In DM, SSc and RA patients, the top 3 elevated tumor markers observed were FRT, HE4 and CYFRA21-3. In SLE patients, the top 3 elevated tumor markers observed were FRT, HE4 and CA125. Notably, with the exception of CA125, the profile of abnormal rates of other tumor markers were comparable in SLE and RA patients. Specifically, the abnormal rate of CA125 in SLE patients was significantly higher than that in RA patients (23.56 vs. 8.13%, *P* < 0.01). In addition, the abnormal rate of CA72-4 and FRT in DM patients was higher than that in the other AID patients.

**Table 2:** Abnormal rate of tumor markers in AID

Tumor marker (reference interval)	Patients with increased tumor maker				χ^2^	P
	DM	SSc	SLE	RA		
CA72-4 (0–6.9 U/mL)	31.06%^abc^	11.69%	19.90%	11.88%	20.43	< 0.01
AFP (0–7 ng/mL)	6.06%	2.60%	2.09%	1.25%	6.79	0.10
FRT (13–150 μg/L)	57.58%^abc^	35.06%	40.31%	36.25%	16.95	< 0.01
NSE (0–16.3 ng/mL)	32.58%^bc^	19.48%	12.04%	11.88%	27.94	< 0.01
CA19-9 (0–27 U/mL)	12.88%c	14.29%c	12.57%	5.00%	7.82	0.05
CA125 (0–35 U/mL)	16.67%^ac^	32.47%^c^	23.56%^c^	8.13%	24.64	< 0.01
CYFRA21-1 (0–3.3 ng/mL)	37.12%bc	33.77%bc	18.85%	16.25%	24.15	< 0.01
CA153 (0–26.4 U/mL)	15.91%bc	15.58%bc	2.62%	2.50%	33.04	< 0.01
BHCG (0–7 U/mL)	3.03%	1.30%	1.57%	1.88%	1.12	0.80
HE4 (0–105.1 pmol/L)	39.39%a	57.14%bc	35.60%	29.38%	17.64	< 0.01
CA50 (< 20 U/mL)	9.85%	7.79%c	5.24%	3.75%	5.27	0.15
SCC (< 1.2 ng/mL)	7.58%	3.90%	5.76%	9.38%	3.06	0.42

### The number of increased tumor markers in AID patients

**Fig. 2:**
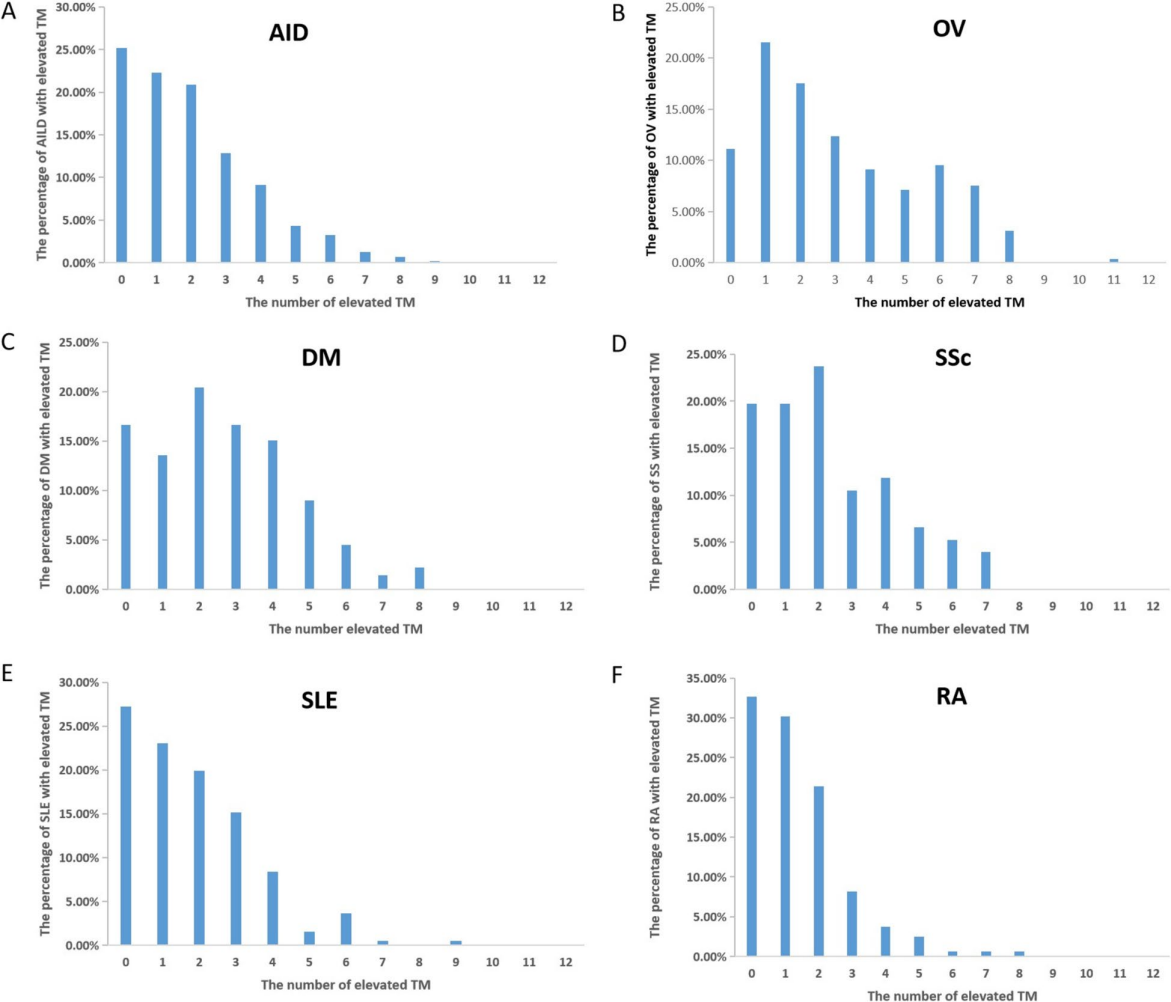
Ratio of patients with different number of increased tumor markers. (A) Ratio of AID patients with different number of elevated TM. (B) Ratio of OV patients with different number of elevated TM. (C-F) Ratio of DM, SSc, SLE and RA with different number of elevated TM. TM: tumor marker

We further investigated the percentage of patients with varying numbers (ranging from 1 to 12) of elevated tumor markers. Specifically, at least one tumor marker was found to be elevated in 88.80% OV patients and 75% AID patients (Fig. 1a, b). Furthermore, 2–5 tumor markers were elevated in 68% of OV patients and 69.46% of AID patients. 6–8 tumor markers were elevated in 20.4% of OV patients and 5.17% of AID patients (Fig. 2a, b). In addition, all the 12 tumor markers were negative in 16.67, 19.74, 27.23 and 32.70% of DM, SSc, SLE and RA patients (Fig. 2c, f).

### The correlation between tumor markers in AID and OV patients

**Fig. 3:**
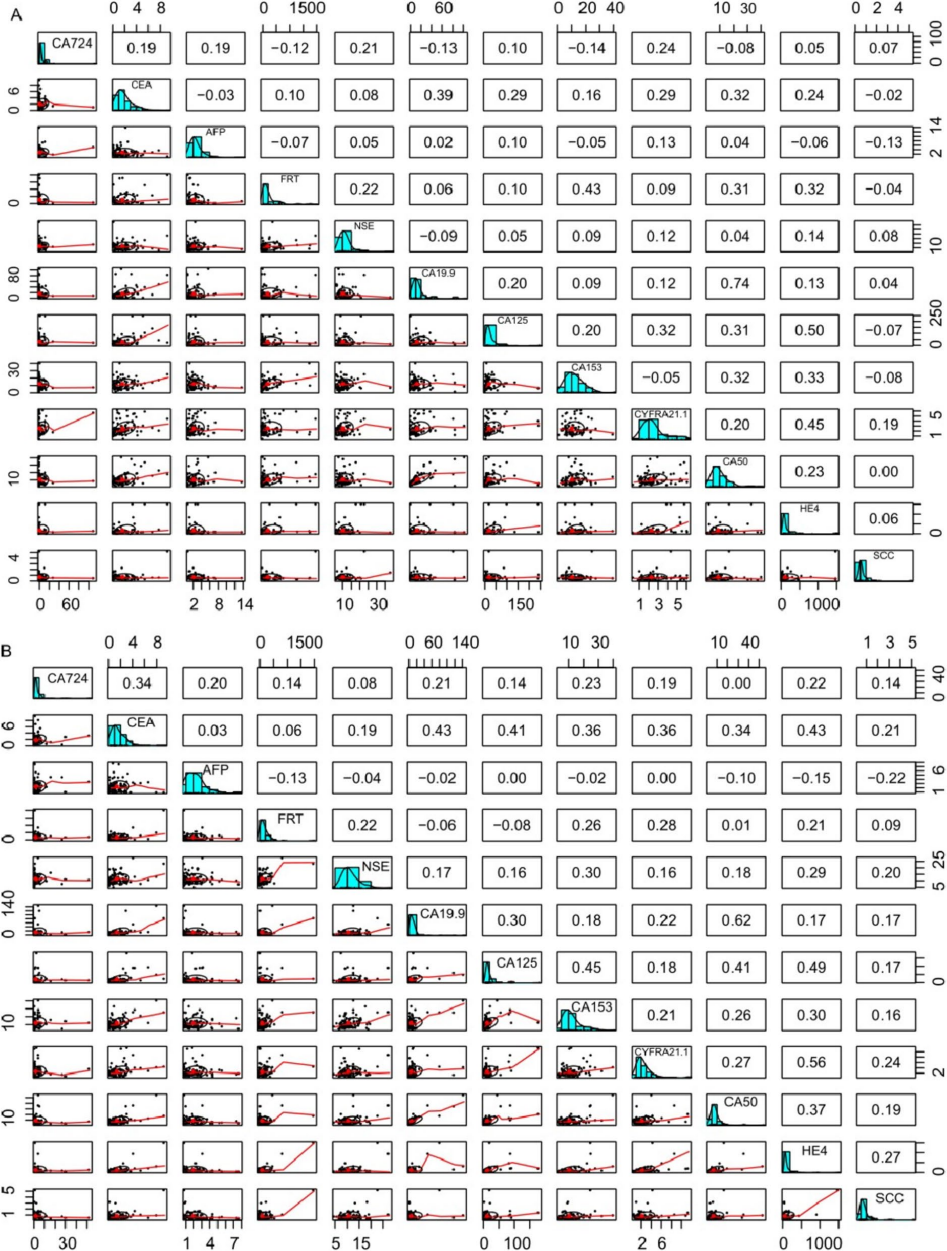
Correlation matrix of tumor makers in AID (A) and OV (B) patients

In AID patients (Fig. 3a), there was a high correlation between CA50 and CA19-9 levels (*r* = 0.75, *P* < 0.001). HE4 was correlated with CA125 (*r* = 0.46, *P* < 0.001) and CYFRA21-1 (*r* = 0.55, *P* < 0.001). There were no significant correlations among the other tumor markers (*r* < 0.4). In OV patients (Fig. 3b), CA50 was high correlated with CA19-9 (*r* = 0.71, *P* < 0.001). HE4 was correlated with CA125 (*r* = 0.65, *P* < 0.001) and CYFRA21-1 (*r* = 0.64, *P* < 0.001). CA153 was correlated with CA125 (*r* = 0.59, *P* < 0.001) and CYFRA21-1 (*r* = 0.43, *P* < 0.001). CA724 was correlated with CA125 (*r* = 0.45, *P* < 0.001).

## Discussion

Autoimmune tolerance is an important part of human immune homeostasis. Consequently, dysregulation of autoimmune mechanisms plays a pivotal role in the pathogenesis and progression of numerous diseases. The interaction between tumorigenesis and autoimmunity has emerged as one of the most prominent research areas in both basic science and clinical practice in recent years. First, a diverse range of autoantibodies can be detected in tumor patients and serve as critical biomarkers for early diagnosis [8, 17], prediction of tumor progression [18], prognosis evaluation [19], and prediction of drug response [20]. Second, patients with autoimmune diseases exhibit an elevated susceptibility to cancer, particularly gynecological malignancies, such as ovarian and breast cancer [21–23], suggesting that patients with autoimmune diseases may also have characteristics or biomarkers similar to cancer patients, or generate factors that promote tumor initiation and progression. Consequently, investigating tumor-associated factors in patients with autoimmune diseases holds substantial significance for the long-term management and prognosis of these patients.

Based on a population study [21], Hill CL et al.’s study demonstrated that dermatomyositis is significantly associated with a wide range of cancers, particularly ovarian cancer [standardized incidence ratios (SIR): 10.5, 95% confidence interval (CI) 6.1–18.1]. Based on Canadian Scleroderma Research Group registry data, Hoa et al.’s study demonstrated that 3.5% of systemic sclerosis (SSc) patients were diagnosed with cancer within a 5-year period. The most prevalent types of cancer diagnosed were breast and gynecological [23]. Notably, SSc patients who tested positive for anti-topoisomerase I and anti-U1-RNP antibodies exhibited a significantly increased risk of developing cancer within a 2-year period. Peng H et al.’s study also indicated an increased risk of lung cancer among SSc patients [24]. For SLE patients, Song L’s meta-analysis demonstrated that SLE was correlated with an elevated risk of 16 involved cancers and decreased risk for prostate cancer and cutaneous melanoma [25]. Recently, Zhou Z’s study showed that the SIR of cancer in SLE, RA, and SSc patients were 2.58 (95% CI 2.07–3.17), 3.99 (95% CI 3.40–4.65) and 3.77 (95% CI 2.49–5.49), respectively. Cancers with elevated SIRs were predominantly observed in females [6]. In summary, accumulating evidence ha suggests an increased susceptibility to cancer among patients with AIDs. Furthermore, elevated SIRs were associated with AID type, cancer type and ethnicity. Notably, several autoantibodies have been identified as potential risk factors for cancer development [22, 26, 27]. However, the tumor marker profiles of patients with AIDs remain inadequately characterized.

This study aimed to investigate the expression profiles of 12 tumor markers in four common autoimmune disease (DM, SSc, SLE, RA), which predominantly affect female. First, a significant proportion of patients with AIDs exhibited serum tumor marker levels exceeding the reference range. Furthermore, no statistically significant differences were observed in the levels or abnormal rates of CYFRA21-1, HE4, CA50, and FRT between AID patients and OV patients. The levels and abnormal rates of CA724, FRT, CA125 and NSE in OV patients were higher than those in AID patients. On the whole, 88.80% OV and 75% AID patients exhibited at least one elevated tumor marker. Furthermore, 2–5 tumor markers were elevated in 68% of OV (68%) and 69.46% of AID patients. Eva Szekanecz et al. demonstrated that the production of CEA, CA19-9, CA125, and CA153 may be increased in patients with RA, scleroderma, lupus, and Sjögren's syndrome (SS) [28]. In addition, Eva Szekanecz et al. reported that a significantly higher proportion of SSc patients exhibited abnormally elevated levels of CA19-9 (8.8 vs. 2.0%), CA125 (11.0 vs. 6.0%) and CA15-3 (28.4 vs. 14.0%) compared to healthy controls. The levels of CEA (32.5 vs. 20.0%), CA19-9 (7.5 vs. 2.0%), CA125 (15.0 vs. 6.0%) and CA72-4 (15.0 vs. 8.0%) were significant elevated in the SLE group compared to the healthy control group [29]. Taken together, these findings indicate that tumor markers expressed in patients with AID exhibit at least partial similarities to those observed in cancer patients.

It is crucial to emphasize that elevated tumor markers in autoimmune diseases (AID) do not necessarily indicate the presence of cancer. In this study, none of the AID patients involved have developed cancer up to now. Bergamaschi S et al.’s study also showed that elevated CEA and CA19-9 are more frequently observed in RA patients, irrespective of an actual cancer diagnosis. [30]. During clinical evaluations, given the unique characteristics of tumor markers, reports of elevated tumor markers may induce psychological stress or potential harm to patients. Therefore, when interpreting tumor marker test results in a clinical context, it is essential to exercise caution regarding elevated values.

In addition, among different type of AID, the profile of tumor markers have significant difference. Specifically, apart from CA50, the other 11 tumor markers exhibited significant differences across DM, SSc, SLE and RA. Furthermore, with the exception of AFP, β-HCG and SCC, the abnormal rates of the other tumor markers were difference between these AIDs. Notably, excluding CA125, the distribution patterns of other were comparable between SLE and RA patients. The negative rates for all 12 tumor markers in DM, SSc, SLE and RA patients were 16.67, 19.74, 27.23 and 32.70%, which means more pronounced impact of DM and SSc on tumor markers profiles. Taken together, these findings demonstrated that there were heterogeneity in tumor marker pattern among patients with different type of AIDs.

In conclusion, this study systematically characterized the tumor marker profiles associated with four autoimmune diseases, including DM, SSc, SLE and RA. The elevated levels of tumor markers were detected in patients with these AIDs. Given the observed elevation in tumor markers and established cancer risk in the AID patients, regular monitoring of tumor markers may serve as a valuable tool for long-term cancer surveillance in this population. However, this study is subject to certain limitations that warrant acknowledgment. First, the sample size was limited. Second, the duration of follow-up was insufficient to comprehensively assess the long-term risk of malignant tumors in AID patients, extended follow-up studies are, therefore, necessary. Third, the study exclusively included female participants, highlighting the need for future investigations to incorporate male patients and conduct gender-based comparative analyses.

## Data Availability

No datasets were generated or analysed during the current study.
